# Supplementary material for: Migration of solidification grain boundaries and prediction
Source: Nat Commun. 2022 Oct 7;13:5910. doi: 10.1038/s41467-022-33482-8 (PMC9547067; doi:10.1038/s41467-022-33482-8)
Supplement: Supplementary file 1 — Supplementary Information [file 41467_2022_33482_MOESM1_ESM.pdf]

Supplementary Information for [Migration of solidification grain boundaries and prediction]

Hongmei Liu[1, 2, 3*], Shenglu Lu[4], Yingbo Zhang[1], Hui Chen[1], Yungui Chen[2], Ma Qian[4*]

[1]*School of Materials Science and Engineering, Southwest Jiaotong University, Chengdu, 610031, China*

[2]*School of Materials Science and Engineering, Sichuan University, Chengdu, 610065, China.*

[3]*Department of Chemical and Materials Engineering, The University of Auckland, Auckland 1010, New Zealand*

[4]*Centre for Additive Manufacturing, School of Engineering, RMIT University, Melbourne, VIC 3000, Australia*

*Corresponding authors: lhm@home.swjtu.edu.cn and ma.qian@rmit.edu.au

1: Sn-enriched interdendritic regions in the Mg-0.63at.%Sn alloy

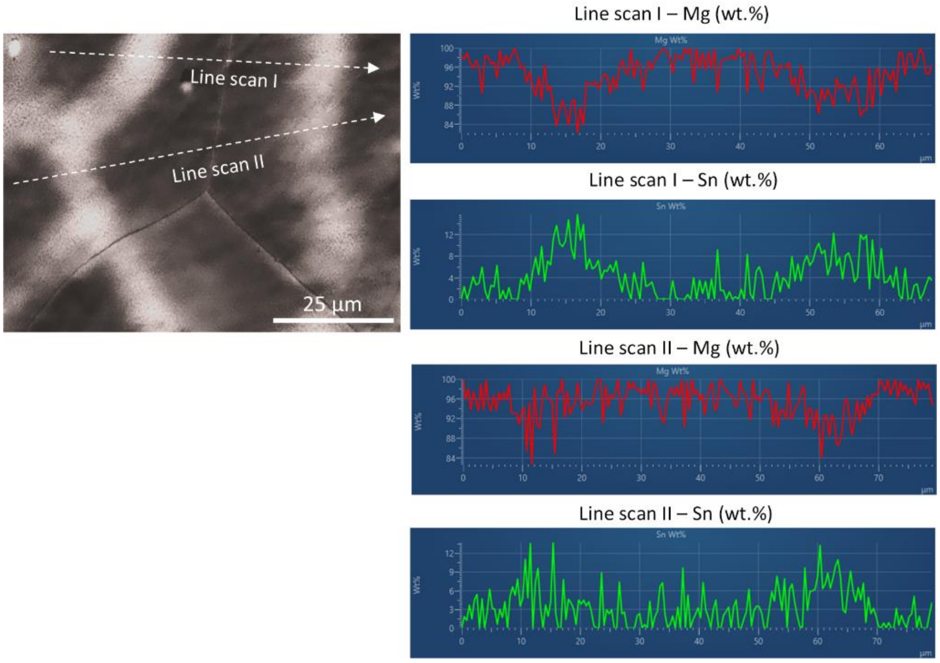

Supplementary Figure 1 Sn-enriched interdendritic regions in the Mg-0.63at.%Sn alloy. Line scans showing the compositional profiles of Mg and Sn across the bright regions and migrated SGBs. The bright regions are confirmed to be Sn-enriched, containing 5-15 wt.%Sn, compatible with the solubility limit of Sn in α-Mg (15 wt.%) at the eutectic temperature.

2. Presence of eutectic α-Mg(Sn)+Mg_2Sn phases in the Mg-1.52at.%Sn alloy (7wt.%)

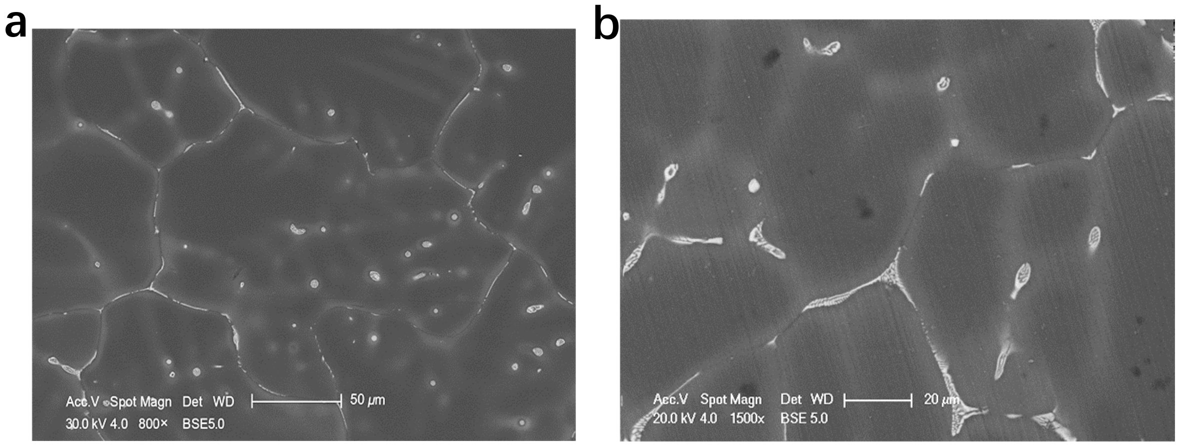

Supplementary Figure 2 Eutectic formation in the Mg-1.52at.%Sn alloy. BSE images showing the formation of noticeable eutectic α-Mg(Sn)+Mg_2Sn structures both within the grains and along the SGBs in the Mg-1.52at.%Sn (7wt.%Sn) alloy in which SGBM still occurred (see Fig. 3 **g** and **h** in the article). **a** and **b** are viewed at two different magnifications.

3. Further examples of localized small SGBM

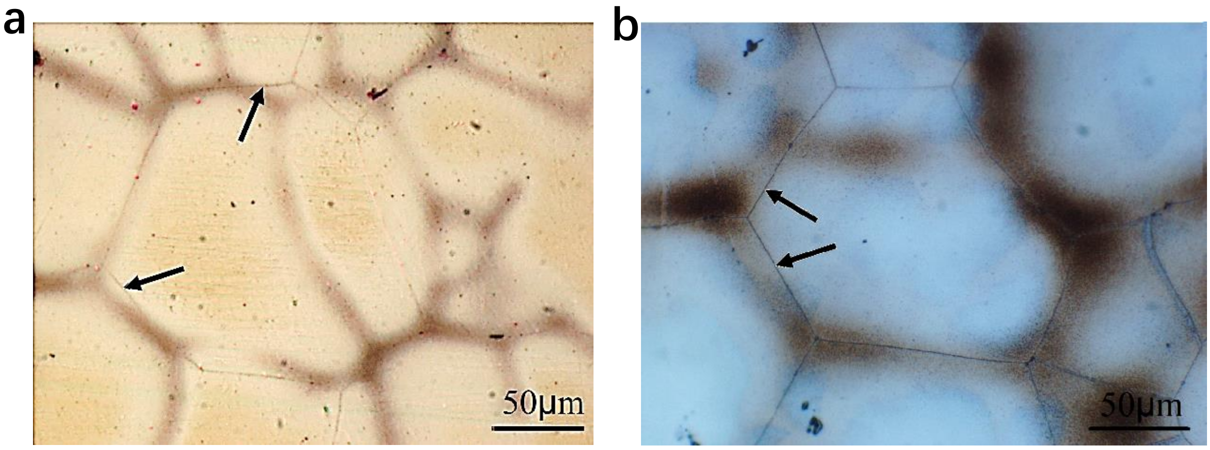

Supplementary Figure 3 Examples of localized SGBM in as-cast magnesium alloys. **a** As-cat Mg-0.3at.%Pb alloy. **b** As-cast Mg-0.3at.%Pb-0.5at.%Zr alloy. In both alloys, SGBs (arrows in **a** and **b**) have only slightly departed from their initial as-solidified positions. After migration, the SGBs are close to their expected equilibrium state (120°-120°-120° triple junctions plus $d/a \approx \sqrt{3}$, where *d* is grain size and *a* grain side length).

4. Elastic strain energy as a source of the driving force for SGBM near the $T_{solidus}$

As pointed out in the main text, Shibata et al[1]. have clarified through specially designed experiments that the strain or stress generated during cooling after solidification has a minor or negligible influence on SGBM. In fact, research has shown that, at typical GBM temperatures (clearly below $T_{solidus}$), the elastic strain energy ($G_{elastic}$) as a source of the driving force for GBM is usually much smaller than the GB energy (G_{GB}) for both micron-sized and nano-sized grains[2-4], namely $G_{elastic}$ is usually negligible when G_{GB} operates. This is in line with the experimental work of Ref. [1].

To further clarify this issue, let us consider an alloy, which has an average grain size of 100 μm, specific SGB energy of 0.5 J/m^2 near $T_{solidus}$ (usually 0.52 – 0.90 J/m^2 at $T_{solidus}$, Supplementary Table 2), and elastic modulus of 100 GPa at $T_{solidus}$ (high for a normal alloy). We then apply a stress of 10 MPa to the alloy at $T_{solidus}$. Note that the strength of most alloys is very small at $T_{solidus}$ (< 1 MPa[5-7]). The stress applied at the magnitude of 10 MPa at $T_{solidus}$ is thus substantial. Using these parameters, Gottstein and Shvindlerman compared $G_{elastic}$ and G_{GB} as driving forces for GBM[2]. $G_{elastic}$ is two orders of magnitude smaller than G_{GB}, i.e., it is negligible[2].

5. The specific grain boundary energy of pure metals (σ_0) at elevated temperatures

The specific grain boundary (GB) energy of pure metals (σ_0) can be estimated using[2]

$$\sigma_0 = \frac{\delta}{b^3} k_B T \quad (1)$$

where δ is the GB thickness, which can be taken as 3-4 atoms wide or ~1 nm in general, b is the atomic diameter of the metal element, k_B is the Boltzmann constant (1.381×10^{-23} J/K) and T is temperature (K). Despite its simple form, the model has shown excellent agreement with experimentally measured average specific GB energy values at high temperatures[1]. Supplementary Table 1 shows the excellent agreement between the experimental data on σ_0 for metals (very limited)[8-10] at high temperatures with their calculated values of σ_0. Supplementary Table 2 lists the specific GB energy values of the five solvent metals studied in this work at elevated temperatures.

Supplementary Table 1 Measured and calculated specific GB energy values of Fe, Cu and Ag at elevated temperatures (σ_0, J/m^2)

Metal	Measured	Calculated using Eq. (1)
Fe	0.79 (1723K) [8]	0.78 (1723K)
Cu	0.70 (1313K) [9]	0.74 (1313K)
Ag	0.46 (1205K) [10]	0.46 (1205K)

Supplementary Table 2 Specific GB energy values of six solvent metals estimated using Eq. (1) at elevated temperatures (σ_0, J/m^2)

Metals	Temperature range (K)	Calculated value
Mg	913-923	0.52 1.60
Al	903-933	0.98 1.
Fe	1780-1810	0.82
Cu	1340-1360	0.77
Ti	1940-1943	0.62
Zr	2130-2133	0.90

6. Assessment of Eqs. (7-9) for the last liquid film composition and the β parameter

Accurate numerical solutions to Eqs. (7-9) are obtainable by simultaneous iteration of ΔT and D_s (D_s is a function of temperature), with the assistance of the liquidus line equation due to the use of the equilibrium solute partition coefficient k in Eq. (7). Supplementary Table 3 lists all the experimental and phase-diagram parameters used for numerically solving Eqs. (7-9) for the 10 dilute alloy systems considered. The solutions are plotted in Supplementary Figure 4 in terms of X_L versus X_0, with f_s being varied from 0.25 to 1.

The consistent perfect linear relationship shown in Supplementary Figure 4 **a-f** for each f_s from 0.25 to 1 and each dilute alloy system, confirms the high self-consistency of the model. Note that the value of k (k < 1) for these 10 alloy systems ranges from 0.06 to 0.78, covering most practical alloys.

Supplementary Table 3 Parameters used to solve Eqs. (7-9) by simultaneous iteration of ΔT and D_s

Alloy system X_0 (at.%)	k	m_l (K/at.%)	ΔT_0 (K)	D_s (m^2/s)	SDAS λ (μm)	Cooling rate $\dot{T}$ (K/s)	Ref. D_s	Ref. λ, $\dot{T}$
Mg-0.3Zn	0.06	-7.35	38.63	$8.7\times10^{-5}exp(-125073/RT)$	45	8	11	*
Mg-0.3Al	0.36	-6.63	3.54	$1.2\times10^{-4}exp(-141814/RT)$	45	8	11	*
Mg-0.3Sn	0.31	-7.38	4.93	$1.1\times10^{-4}exp(-140925/RT)$	45	8	11	*
Mg-0.3Pb	0.41	-9.63	4.16	$6.5\times10^{-6}exp(-119400/RT)$	45	8	12	*
Al-0.3Mg	0.49	-9.05	2.82	$1.5\times10^{-5}exp(-120500/RT)$	35	10	13	14
Al-0.3Cu	0.15	-7.03	11.95	$4.4\times10^{-5}exp(-133900/RT)$	35	10	13	15
Cu-0.3Sn	0.44	-11.96	7.68	$8.2\times10^{-5}exp(-187600/RT)$	45	20	16	17
Fe-0.3C	0.16	-15.03	24.01	$1.3\times10^{-6}exp(-81398/RT)$	14	65	18	18
Ti-0.3Cr	0.71	-9.06	1.16	$1.8\times10^{-6}exp(-168500/RT)$	25	120	19	20
Zr-0.3Mo	0.78	-6.65	0.63	$2.1\times10^{-4}exp(-286800/RT)$	30	0.2	16	21

*: experimentally measrued (this study)

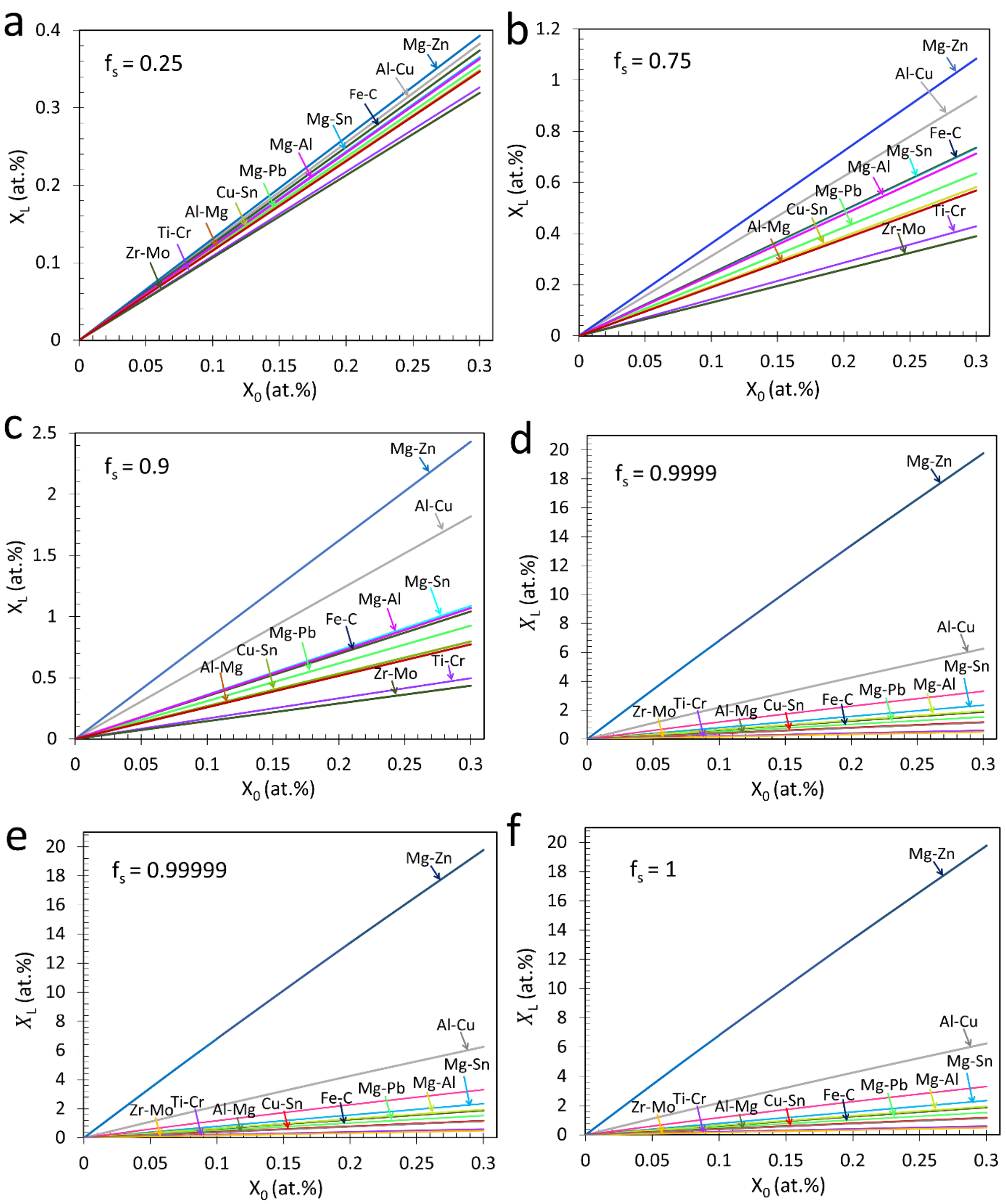

Supplementary Figure 4 Predictions of the remaining liquid composition (X_L) for 10 alloy systems. The predictions are obtained from solving Eqs. (7-9) in the article via simultaneous iteration of both ΔT and D_s using the parameters listed in Supplementary Table 3. The solid fraction f_s is varied from 0.25 to 1. **a** $f_s = 0.25$, **b** $f_s = 0.75$, **c** $f_s = 0.9$, **d** $f_s = 0.9999$ (~10 nm thick liquid film left), **e** $f_s = 0.99999$ (~1 nm thick liquid film left), and **f** $f_s = 1$ (complete solidification).

Based on the numerical solutions presented in Supplementary Figure 4 **a-f** and the data listed in Supplementary Table 3, we first assessed the β value for these 10 dilute alloy systems at a selected experimental cooling rate for each system (see Supplementary Table 3). The solute concentration is varied from 0.01 at.% to 0.3 at.%. The results are summarized in Supplementary Table 4. The value of β varies in a very narrow range for each dilute alloy system assessed (see the STDEV in Supplementary Table 4). The underlying reason can be attributed to the fact that increasing X_0 increases ΔT but decreases D_s. Consequently, their influences largely cancel out. Hence, for dilute alloys, at similar cooling rates, the value of α+ calculated from Eq. (9) only varies in a narrow range. On the other hand, β is not highly sensitive to a small variation of α^+. As a result, β varies in a very narrow range for each of the 10 dilute binary alloy systems assessed.

Supplementary Table 4 The values of β calculated from Eqs. (7-9) by simultaneous iteration of ΔT and D_s

X_0 (at.%)	Alloy system and the β parameter									
	Mg-Sn	Mg-Pb	Mg-Al	Mg-Zn	Fe-C	Al-Cu	Ti-Cr	Cu-Sn	Zo-Mo	Al-Mg
0.01	0.194	0.194	0.194	0.195	0.964	0.197	0.194	0.197	0.196	0.196
0.1	0.195	0.194	0.194	0.197	0.981	0.200	0.197	0.202	0.199	0.199
0.2	0.197	0.195	0.195	0.200	0.990	0.207	0.201	0.209	0.204	0.205
0.3	0.199	0.196	0.196	0.203	0.994	0.213	0.204	0.216	0.209	0.211
Mean	0.196	0.195	0.195	0.199	0.982	0.204	0.199	0.206	0.202	0.203
STDEV	0.00293	0.00135	0.00148	0.00424	0.01050	0.00843	0.00413	0.00965	0.00596	0.00740

We then systematically assessed the dependence of β on cooling rate for different alloys. The results are listed in Supplementary Table 5. β is effectively independent of the wide range of cooling rates assessed for each alloy (up to 2160 K/s, covering most normal casting processes). Also listed in Supplementary Table 5 are the assessments of the effect of cooling rate on the actual solidification range ΔT. Akin to its influence on β, the influence of cooling rate on ΔT is negligible, which agrees well with the assessment of Won and Thomas[8]. Similarly, the underlying reason is thatt, for a given dilute alloy, increasing $\dot{T}$ decreases the SDAS λ (λ and $\dot{T}$ are correlated[18,22]). Again, their influences largely cancel out in Eq. (9) for the calculation

of α^+, while β is not highly sensitive to a small variation of α^+. Their combined effects result in β being essentially independent of cooling rate.

The above systematic assessments indicate that β can be effectively regarded as a constant for each dilute binary alloy system assessed. To validate this finding, Supplementary Figure 5 **a-f** compares our accurate numerical solutions to Eqs. (7-9) obtained by simultaneous iteration of ΔT and D_s with those obtained from solving Eq. (7) with a constant value of β listed in Supplementary Table 4. The match is perfect for each of the 10 dilute binary alloy systems.

Supplementary Table 5 Effect of cooling rate ($\dot{T}$) on the secondary dendrite arm spacing (λ), actual solidification temperature gap ΔT, and value of β for different alloys

Alloy (at.%)	SDAS (λ, μm)	Cooling rate ($\dot{T}$, K/s)	ΔT (K)	β	Mean value of β	STDEV of β
Mg-0.63Sn	41.6 ± 14.8	~8	15.1	0.204	0.205	0.0015
Mg-0.63Sn	18.2 ± 5.4	~54	15.0	0.207		
Mg-0.63Sn	11.1 ± 3.7	~166	15.0	0.206		
Mg-0.63Sn	4.0 ± 1.4	~1690	15.1	0.203		
Mg-2.67Al	40.1	6	93.3	0.212	0.206	0.0045
Mg-2.67Al	13.5	80	95.1	0.206		
Mg-2.67Al	8.8	220	95.4	0.205		
Mg-2.67Al	3.4	2160	96.6	0.201		
Al-0.41Cu	48.5	12	67.6	0.195	0.195	0.0005
Al-0.41Cu	24.2	58	67.6	0.195		
Al-0.41Cu	15.7	155	67.6	0.195		
Al-0.41Cu	5.2	1860	67.8	0.194		
Fe-0.0095C[8]	137.4	1	47.37	Data from Won and Thomas [18]		
Fe-0.0095C[8]	44.1	10	47.44			
Fe-0.0095C[8]	14.2	100	47.52			

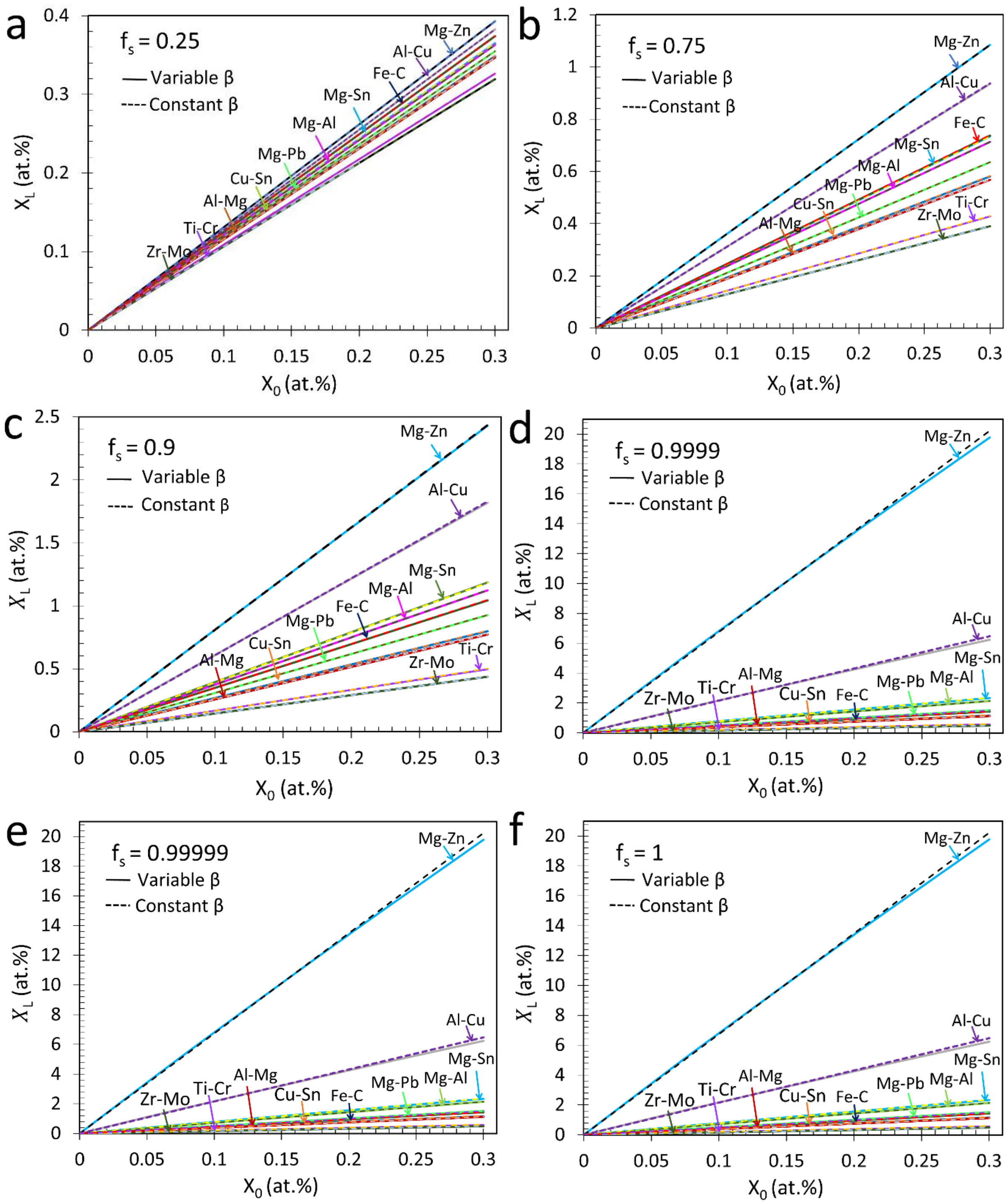

Supplementary Figure 5 Predictions of the remaining liquid composition (X_L) using two methods. The solid lines indicate the "Variable β" approach, obtained by solving Eqs. (7-9) via simultaneous iteration of ΔT and D_S. The dashed lines indicate the "Constant β" approach, obtained by solving Eq. (7) with a mean value of β (Supplementary Table 4). The predictions are almost identical. **a** f_s = 0.25, **b** f_s = 0.75, **c** f_s = 0.9, **d** f_s = 0.9999 (~10 nm thick liquid film left), **e** f_s = 0.99999 (~1 nm thick liquid film left), and **f** f_s = 1 (complete solidification).

7. Solidification grain boundary (SGB) atomic density ρ in dilute binary alloys

For low angle (θ) GBs, the relative GB atomic density can be estimated using a proposed analytical model [1 – sin (θ)/4][23]. However, SGBs are typically high angle GBs[24,25], whose atomic density is estimated to be about 5%-15% less than that of the bulk grains[26]. In their treatment of the Ni-3.6at.%P alloy, where the GB contains up to 15at.%P (P is an interstitial segregating element at temperatures of practical importance[27]), based on tomographic atom probe (TAP) data, Liu and Kirchheim[28] used the atomic density of Ni to estimate the GB atomic density.

The use of Eq. (11) to define the last liquid film composition (X_L), i.e., the average composition of a SGB (X_i^{Φ}) immediately after solidification, makes the determination of the SGB atomic density much easier. We have calculated X_L (i.e. X_i^{Φ}) in dilute binary alloys containing up to 0.5at.% of the solute. Its value is generally less than or around 5at.%, e.g., 3.82at.% for Mg-0.5at.%Pb, 5.30at.% for Mg-0.5at.%Al, 4.72at.% for Mg-0.5at.%Sn, and 2.81at.%C for Fe-0.5at.%C. The GB atomic density (ρ_{GB}) refers to the number of moles of all atoms per unit volume of the GB. Given the above estimates, to keep our final analytical model simple, following Ref. [28], we use the solvent atomic density as an estimate of the ρ_{GB} in our calculations. This is equivalent to assuming that the change in the volume of 1 mole of the molten solvent atoms is generally negligible after introducing 5at.% of a solute species at the same temperature.

8. Raw experimental data or measurements

Supplementary Table 6 SDAS λ and grain size *d* for Mg-(0.21-1.07)at.%Sn alloys at the cooling rate of 8 °C/s

Measurements	SDAS λ (μm) and grain size *d* (μm) at the cooling rate of 8 °C/s							
	Mg-0.21at%Sn		Mg-0.30at%Sn		Mg-0.42at%Sn		Mg-1.07at%Sn	
	SDAS	Grain size	SDAS	Grain size	SDAS	Grain size	SDAS	Grain size
1	29.7	150.7	57.5	240.9	41.8	148.0	30.8	93.9
2	41.1	270.9	26.4	134.2	26.0	121.2	25.8	123.2
3	46.2	307.8	40.9	271.0	41.2	216.9	40.9	130.2
4	59.8	283.2	64.8	150.6	48.0	243.9	19.7	128.8
5	25	153.8	21.2	251.8	66.1	226.7	47.7	70.2
6	59.2	236.9	20.4	136.9	19.8	219.4	65.6	100.9
7	49.9	169.2	51.3	210.8	52.0	123.6	51.6	78.6
8	23.5	215.4	39.4	191.7	22.6	177.6	22.5	84.2
9	61.6	273.9	31.5	243.6	21.1	135.9	41.5	124.6
10	26.5	236.9	35	210.8	35.1	189.9	34.8	107.9
11	45.8	304.8	37.7	150.6	57.4	243.9	51.1	124.6
12	26.7	172.5	46.9	271.0	23.2	202.2	47.1	73.1
13	67.1	230.8	22.6	153.4	68.8	113.8	23.0	105.1
14	24.5	240.1	73.9	205.3	45.7	185.1	69.5	109.3
15	58.6	252.4	20.5	213.5	37.8	192.4	37.6	114.9
16	47.9	320.2	45.3	224.5	50.3	231.7	49.9	131.6
17	27.1	169.2	49.7	257.3	22.6	123.6	22.5	84.2
18	17.6	181.5	23.1	161.6	24.8	219.4	41.5	82.8
19	61.6	273.9	41.5	243.6	41.8	145.7	24.7	124.6
20	27.7	267.8	62.1	238.1	63.2	214.5	62.8	121.8
21	26.9	240.1	35.6	213.5	35.6	192.4	35.3	109.3
22	47.1	163.0	58.6	265.5	21.5	131.0	39.8	74.4
23	49.4	258.5	28.8	145.2	39.0	229.2	21.3	117.6
24	19.1	264.7	20.9	229.9	30.1	207.1	38.7	123.2
25	54.5	317.1	55.3	235.4	55.8	212.1	51.1	130.2
26	28.2	249.3	39.9	254.6	37.3	199.7	37.0	113.5
27	45	298.6	27.2	221.7	46.3	239.1	45.9	128.8
28	20.3	264.7	45.8	235.4	60.1	212.1	39.8	120.4
29	61.2	298.6	41.1	265.5	31.2	239.0	46.5	127.4
30	72.6	292.4	71.1	260.0	68.7	236.6	65.6	119.1
31	68.1	295.5	62.6	262.8	63.8	234.1	63.3	116.3
32	25.4	172.3	46.4	153.4	23.2	118.7	23.0	75.8
33	30.1	190.7	26.9	169.8	26.6	153.1	68.9	100.9
34	41.1	240.1	51.0	213.5	31.2	192.4	26.4	109.3
35	25.8	159.9	75.8	142.4	20.9	123.7	49.9	70.2
36	27.3	212.3	21.5	188.9	36.2	170.3	35.3	89.8

37	23.7	246.2	46.1	219.0	36.7	204.6	20.8	116.3
38	62.9	289.3	26.7	257.3	50.3	167.8	30.3	131.6
39	70.9	209.2	49.6	186.2	69.5	231.7	36.5	95.3
40	48.6	252.4	27.7	224.5	65.6	202.2	37.6	114.9
41	65.8	286.3	54.5	254.6	59.3	229.2	54.9	116.3
42	22.4	280.1	74.5	249.1	42.9	224.3	42.6	127.4
43	67.5	277.2	45.6	199.9	64.9	180.1	64.5	128.8
44	52.8	258.5	46.9	251.8	47.4	207.1	31.9	117.6
45	30	190.6	26.9	229.9	26.6	160.5	47.1	105.1
46	67.1	243.1	59.9	183.5	64.3	194.8	26.3	110.7
47	24.3	221.6	74.0	216.3	32.2	182.6	63.9	103.7
78	43.5	215.4	22.2	202.6	31.1	172.7	30.9	98.1
49	22.6	209.3	31.2	191.7	29.9	167.8	29.7	95.3
50	56.0	224.6	30.1	186.2	33.8	226.7	60.5	102.3

Supplementary Table 7 SDAS λ and *d* grain size (μm) for Mg-0.63at.%Sn alloy at different cooling rates (°C/s)

Measurements	SDAS (μm) λ and grain size *d* (μm) for Mg-0.63at.%Sn alloy at different cooling rates (°C/s)							
	8 (°C/s)		54 (°C/s)		166 (°C/s)		1690 (°C/s)	
	SDAS	Grain size	SDAS	Grain size	SDAS	Grain size	SDAS	Grain size
1	39.9	124.2	14.0	62.9	13.9	66.9	3.1	36.1
2	41	167.3	22.7	84.8	8.5	53.5	5.0	46.1
3	42.7	157.1	24.8	79.6	14.9	71.1	5.5	43.7
4	23.1	175.5	24.4	87.4	15.1	47.7	5.4	47.3
5	46.1	99.6	7.9	52.1	9.1	73.2	1.6	30.9
6	22.6	128.4	16.1	65.0	4.2	44.8	3.3	37.0
7	47.2	101.7	9.4	54.1	5.7	55.2	2.1	31.9
8	20.5	109.9	11.1	55.7	14.1	60.2	2.4	32.7
9	47.8	169.4	23.2	85.8	6.8	46.4	5.1	46.7
10	20.1	144.8	18.2	73.3	11.1	71.9	4.0	40.9
11	65.8	169.4	23.2	85.8	4.7	61.9	5.1	46.6
12	21.4	96.6	7.7	48.9	14.1	46.9	1.7	29.5
13	47.2	140.7	17.3	71.3	10.6	71.9	3.8	39.9
14	22.6	126.3	18.6	64.0	6.2	42.3	2.3	36.5
15	26.5	155.0	20.3	78.5	12.3	54.4	4.5	43.2
16	27.7	179.6	23.2	91.0	6.8	66.1	5.6	49.0
17	28.5	109.9	11.1	55.7	15.4	76.1	2.4	32.7
18	29.8	107.9	10.7	54.6	6.5	47.7	2.3	32.2
19	65.8	179.6	23.2	91.0	16.7	76.1	6.1	49.1
20	69.1	165.3	22.3	83.7	13.6	70.3	4.9	45.7
21	35.4	167.3	18.6	84.8	13.9	71.1	5.0	46.1
22	69.7	95.5	8.2	52.0	5.0	44.8	1.8	31.0
23	30.4	159.1	21.1	80.6	12.9	67.7	4.6	44.2
24	51.2	167.3	22.7	84.8	13.9	73.6	4.9	46.1
25	30.9	177.6	24.8	89.9	15.1	71.1	5.5	48.6
26	31.0	153.0	19.8	77.5	12.1	75.2	4.4	42.8
27	31.9	175.5	24.4	88.9	16.2	65.2	5.8	48.1
28	35.5	181.7	21.9	84.2	16.4	74.4	5.9	45.9
29	50.1	173.5	24.0	87.9	5.2	56.1	5.3	47.6
30	51.3	120.2	21.5	60.9	14.6	70.7	2.9	35.1
31	36.6	157.1	20.7	79.6	8.0	51.9	4.5	43.7
32	51.3	97.6	8.6	54.1	12.6	66.9	1.9	31.9
33	37.1	130.4	16.1	66.1	9.8	46.4	3.3	37.5
34	50.1	153.0	18.6	77.5	11.3	65.2	4.4	42.8
35	37.7	95.5	7.2	55.2	4.2	47.3	1.5	32.4
36	39.9	118.1	12.7	59.8	7.8	71.5	2.8	34.6

37	48.6	157.1	20.7	79.6	12.6	51.0	4.5	43.7
38	51.8	179.6	25.3	85.3	8.8	66.9	5.5	46.4
39	60.7	126.3	14.4	64.0	15.4	54.4	3.2	36.5
40	55.1	155.0	20.2	78.5	12.3	66.1	4.5	43.2
41	63.0	157.1	20.7	79.6	14.6	73.6	4.6	43.7
42	23.1	173.5	24.0	87.9	12.6	66.9	5.3	47.6
43	46.7	175.5	24.4	88.9	15.4	74.4	5.4	48.1
44	24.8	183.7	21.1	89.4	14.9	48.5	5.7	48.3
45	63.5	161.2	17.3	81.6	13.1	74.9	4.7	44.7
46	25.9	112.0	19.0	56.7	6.5	68.6	2.3	33.1
47	64.1	132.5	16.9	67.1	10.3	58.6	3.4	37.9
78	64.7	130.4	15.2	66.1	9.3	56.9	3.4	37.5
49	26.4	126.3	14.4	64.0	10.1	56.1	3.2	36.5
50	37.7	136.6	16.5	69.2	8.8	54.4	3.6	38.9

Supplementary Table 8 Grain side length *a* before and after SGBM for Mg-(0.21-1.07)at.% Sn alloys

Grain		Grain side length *a* (μm)							
		Mg-0.21at%Sn		Mg-0.42at%Sn		Mg-0.63at%Sn		Mg-1.07at%Sn	
	Grain side	Before SGBM	After SGBM	Before SGBM	After SGBM	Before SGBM	After SGBM	Before SGBM	After SGBM
Grain 1	A	280	216	214	191	145	133	112	108
	B	247	182	181	155	167	145	64	64
	C	169	138	263	220	88	76	80	80
	D	292	255	225	161	170	123	96	91
	E	138	124	178	159	187	165	63	63
	F								
Grain 2	A	179	132	128	95	111	102	66	66
	B	198	174	113	83	91	86	84	84
	C	97	83	141	119	102	99	71	61
	D	84	49	102	82	75	71	83	52
	E			92	80	60	52	65	45
	F			57	44				
Grain 3	A	228	211	192	177	105	99	97	72
	B	122	108	169	145	40	37	68	53
	C	190	162	253	218	73	70	105	61
	D	266	242	187	169	68	65	77	77
	E	258	188	188	154	61	60	90	90
	F					45	42	84	84
Grain 4	A	228	204	132	100	133	132	103	103
	B	157	134	170	124	142	105	95	88
	C	202	187	191	145	143	130	90	74
	D	310	184	155	120	75	55	55	51
	E	295	95	127	102	92	71	66	66
	F							84	45
Grain 5	A	165	140	139	106	153	114	87	87
	B	285	234	124	95	98	93	82	82
	C	98	77	150	134	137	116	48	48
	D	203	179	129	112	115	102	65	65
	E	292	280	151	128	178	142	83	83
	F					95	90	45	45
Grain 6	A	296	281	211	187	161	143	84	84
	B	222	187	147	110	140	137	63	63
	C	295	265	105	88	154	140	73	73
	D	166	143	131	122	86	75	75	75

	E<br>F	88	74	85	70	92	90	58	58
Grain 7	A<br>B<br>C<br>D<br>E<br>F	195<br>207<br>287<br>302<br>110	169<br>183<br>197<br>293<br>92	123<br>187<br>201<br>125<br>156	102<br>141<br>154<br>115<br>120	114<br>121<br>103<br>65<br>52	106<br>115<br>98<br>55<br>50	75<br>82<br>71<br>66<br>50	75<br>82<br>71<br>66<br>50
Grain 8	A<br>B<br>C<br>D<br>E<br>F	256<br>187<br>189<br>332<br>189	201<br>146<br>135<br>213<br>158	193<br>142<br>105<br>192<br>115	180<br>130<br>98<br>175<br>110	110<br>107<br>73<br>108<br>56	103<br>105<br>69<br>97<br>54	95<br>71<br>65<br>69<br>80<br>56	55<br>41<br>38<br>61<br>70<br>56
Grain 9	A<br>B<br>C<br>D<br>E<br>F	181<br>235<br>165<br>132<br>295	125<br>165<br>96<br>111<br>254	111<br>196<br>126<br>113<br>98	91<br>152<br>121<br>112<br>80	103<br>109<br>116<br>95<br>147<br>59	94<br>99<br>98<br>90<br>143<br>55	77<br>86<br>65<br>80<br>72<br>51	71<br>78<br>62<br>80<br>72<br>51
Grain 10	A<br>B<br>C<br>D<br>E<br>F	197<br>185<br>307<br>243	123<br>144<br>292<br>211	102<br>205<br>172<br>154<br>165	95<br>190<br>166<br>139<br>154	90<br>142<br>113<br>75<br>101	82<br>134<br>95<br>66<br>85	48<br>59<br>73<br>56<br>66<br>85	48<br>51<br>69<br>56<br>66<br>85

Supplementary Table 9 Grain side length *a* before and after SGBM for Mg-0.3at.%X (X = Zn, Al, Sn, Pb) alloys

Grain		Grain side length *a* (μm)							
		Mg-0.3at%Zn		Mg-0.3at%Al		Mg-0.3at%Sn		Mg-0.3at%Pb	
	Grain side	Before SGBM	After SGBM	Before SGBM	After SGBM	Before SGBM	After SGBM	Before SGBM	After SGBM
Grain 1	A	131	131	166	154	254	233	151	137
	B	77	77	249	218	175	149	149	115
	C	97	97	207	202	190	177	138	106
	D	110	110	139	127	139	133	75	68
	E	75	75			87	72	83	54
	F								
Grain 2	A	101	101	118	116	169	135	229	208
	B	128	128	124	123	207	162	187	150
	C	93	93	131	131	244	176	288	232
	D	80	80	109	109	213	204	245	221
	E	69	69	167	167	133	106	253	206
	F								
Grain 3	A	148	148	120	117	224	198	283	217
	B	111	111	98	94	165	138	192	148
	C	126	126	81	80	147	127	235	180
	D	157	157	117	114	262	194	277	213
	E	183	183	62	51	152	134	188	144
	F				54				
Grain 4	A	117	117	115	114	146	108	167	132
	B	101	101	123	123	215	159	185	148
	C	81	81	114	114	145	124	152	117
	D	64	64	72	72	112	102	164	131
	E	76	76	57	57	187	161	206	157
	F								
Grain 5	A	115	115	177	171	139	131	208	196
	B	92	92	154	143	185	177	159	103
	C	78	78	171	152	230	208	144	137
	D	61	61	95	90	189	182	130	111
	E	74	74	101	92			96	61
	F								
Grain 6	A	172	172	158	149	257	214	162	145
	B	95	95	101	95	224	178	220	197
	C	113	113	142	137	226	182	247	223
	D	79	79	119	114	270	208	188	171

	E	82	82	184	163	166	142	161	146
	F								
Grain 7	A	92	92	149	143	143	114	145	136
	B	100	100	158	154	156	128	228	205
	C	87	87	160	159	109	101	171	162
	D	81	81	86	85	93	76	125	119
	E	62	62	105	103	51	47	157	149
	F								
Grain 8	A	86	86	121	119	220	194	232	185
	B	61	61	89	89	175	127	183	146
	C	54	54	87	87	231	190	140	112
	D	96	96	92	90	236	205	252	201
	E	108	108	74	65	268	171	148	114
	F				47				
Grain 9	A	77	77	121	120	190	162	114	95
	B	90	90	99	99	174	139	217	181
	C	62	62	111	111	197	166	203	169
	D	89	89	82	80	233	152	151	126
	E	57	57	66	66	211	99		
	F	48	48						
Grain 10	A	150	150	143	132	162	123	272	203
	B	121	121	173	161	209	164	235	172
	C	102	102	93	90	124	116	281	220
	D	142	142	167	148	166	157	296	243
	E	135	135	198	172	221	204	193	138
	F								

Supplementary References

1. Shibata, S., Watanabe, T., Mekaru, S. & Fukumoto, I. Effect of various factors on grain boundary migration in the weld metal of an austenitic stainless steel. *J. Jpn. Weld. Soc.* **15**, 100-107 (1997) (in Japanese).
2. Gottstein, G. & Shvindlerman, L. S. *Grain boundary migration in metals: thermodynamics, kinetics, applications. 2nd edn*, (ed. Ralph, B.), 144, 118-119 (CRC Press, Taylor & Francis Group, Boca Raton, 2010).
3. Saxena, R., Cho, W., Rodriguez, O., Gill, W. N. & Plawsky, J. L. Stability of thin copper films on mesoporous dielectrics. *J. Non-cryst Solids* **350**, 14-22 (2004)
4. Gianola, D. S., Petegem S. V., Legros, M., Brandstetter, S., Swygenhoven, H. V. & Hemker,

K. J. Stress-assisted discontinuous grain growth and its effect on the deformation behavior of nanocrystalline aluminum thin films. *Acta Mater.* **54,** 2253–2263 (2006).

5. Eskin, D. G., Suyitno & Katgerman, L. Mechanical properties in the semi-solid state and hot tearing of aluminium alloys. *Prog. Mater. Sci.*, **49**, 629–711 (2004)
6. Summers, P. T., Chen, Y., Rippe, C. M., Allen, B., Mouritz, A. P., Case, S. W. & Lattimer, B. Y. Overview of aluminum alloy mechanical properties during and after fires. *Fire Sci. Rev.* **4**, 3 (2015).
7. Xi, X., Li, S., Yang, S., Li, J. & Zhao, M. Effect of adding yttrium on precipitation behaviors of inclusions in E690 ultra high strength offshore platform steel. *High Temp. Mat. PR-ISR* **39**, 510-519 (2020).
8. Seah, M. P. & Hondros, E. D. Grain boundary segregation. *Proc. B. Soc. Bond.* **A335**, 191-212 (1973).
9. Hondros, E. D. & McLean, D. Cohesion margin of copper. *Philos. Mag.* **29,** 771-778 (1974).
10. Buttner, F. H., Funk, E. R.& Udin, H. Adsorption of oxygen on silver. *J. Phys. Chem.* **56,** 657–660 (1952).
11. Zhong, W. *Measurement of Diffusion Coefficients of Nine Elements in Magnesium and Establishment of a Comprehensive Mobility Database for Lightweight Magnesium Alloys.* Ph.D. Thesis, 96 (The Ohio State University, Columbus, Ohio, 2019).
12. Zhou, B., Shang, S., Wang, Y. & Liu, Z. Diffusion coefficients of alloying elements in dilute Mg alloys: A comprehensive first-principles study. *Acta Mater.* **103,** 573–586 (2016).
13. Du, Y., Chang, Y. A., Huang, B., Gong, W., Jin, Z., Xu, H., Yuan, Z, Liu, Y, He, Y.& Xie, F. Y. Diffusion coefficients of some solutes in fcc and liquid Al: critical evaluation and correlation. *Mater. Sci. Eng.* **A363,** 140-151 (2003).
14. Paliwal, M.& Jung, I. The evolution of the growth morphology in Mg–Al alloys depending on the cooling rate during solidification. *Acta Mater.* **61**, 4848–4860 (2013).
15. Talamantes-Silva, M. A., Rodríguez, A., Talamantes-Silva, J., Valtierra, S.& Colás, R. Characterization of an Al–Cu cast alloy. *Mater. Charact.* **59,** 1434 – 1439 (2008).
16. Neumann, G. & Tuijn, C. *Self-Diffusion and Impurity Diffusion in Pure Metals: Handbook of Experimental Data.* 52, 168 (Oxford, Elsevier, 2009)
17. Kumoto, E. A., Alhadeff, R. O. & Martorano, M. A. Microsegregation and dendrite arm coarsening in tin bronze. *Mater. Sci. Tech.* **18,** 1001-1006 (2002).

18. Won,Y. M. & Thomas, B. G. Metall. Simple model of microsegregation during solidification of steels. *Mater. Trans. A.* **32**, 1755–1767 (2001).
19. Zhu, L., Zhang, Q., Chen, Z., Wei, C., Cai, G., Jiang, L., Jin, Z.& Zhao, J. Measurement of interdiffusion and impurity diffusion coefficients in the bcc phase of the Ti-X (X = Cr, Hf, Mo, Nb, V, Zr) binary systems using diffusion multiples. *J Mater. Sci.* **52,** 3255-3268 (2017).
20. Tedman-Jones, S. N., McDonald, S. D., Bermingham, M. J., StJohn, D. H. & Dargusch, M. S. Investigating the morphological effects of solute on the β-phase in as-cast titanium alloys. *J. Alloys and Compd.* **778**, 204-214 (2019).
21. Suyalatu, Nomura, N., Oya, K., Tanaka, Y., Kondo, R., Doi, H., Tsutsumi, Y. & Hanawa, T. Microstructure and magnetic susceptibility of as-cast Zr–Mo alloys. *Acta Biomater.* **6**,1033-1038 (2010).
22. Dutta, B., & Rettenmayr, M. Effect of cooling rate on the solidification behaviour of Al–Fe–Si alloys. *Mater. Scie. Eng. A* **283,** 218–224 (2000).
23. Kamachali, R. D. A model for grain boundary thermodynamics. *RSC Advances*. **10**, 26728-26741 (2020).
24. Lippold, J. C. & Kotecki, D. J. *Welding Metallurgy and Weldability of Stainless Steels*, (Hoboken, NJ, John Wiley & Sons, 2005)
25. Mustafi, L., Nguyen, V.T., Lu, S.L., Song, T., Murdoch, B.J., Fabijanic, D.M. & Qian, M., 2021. Microstructure, tensile properties and deformation behaviour of a promising bio-applicable new Ti35Zr15Nb25Ta25 medium entropy alloy (MEA). *Mater. Sci. Eng. A*, **824**, 141805 (2021).
26. Priester, L. *Grain Boundaries: from Theory to Engineering.* 156 (Springer, 2013).
27. Lejček, P. & Hofmann, S. Interstitial and substitutional solute segregation at individual grain boundaries of α-iron: Data revisited. *J. Phys. Condens. Matter.* **28**, 064001:1-9 (2016).
28. Liu, F. & Kirchheim, R. Nano-scale growth inhibited by reducing grain boundary energy through solute segregation. *J. Cryst. Growth.* **264**, 385-391 (2004).
